# Spatiotemporal epidemiology of, and factors associated with, the tuberculosis prevalence in northern China, 2010–2014

**DOI:** 10.1186/s12879-019-3910-x

**Published:** 2019-04-30

**Authors:** Xuemei Wang, Shaohua Yin, Yunpeng Li, Wenrui Wang, Maolin Du, Weidong Guo, Mingming Xue, Jing Wu, Danyan Liang, Ruiqi Wang, Dan Liu, Di Chu

**Affiliations:** 1 School of Public Health, Inner Mongolia Medical University, Hohhot, 010110 China; 2 School of Public Health, Peking University, Beijing, 100191 China; 3 Inner Mongolia Ecology and Agrometeorology Center, Hohhot, 010110 China; 4 Inner Mongolia Center for Disease Control and Prevention, Hohhot, 010031 China; 5 School of Basic Medicine, Inner Mongolia Medical University, Hohhot, 010110 China; 6 National Center for Chronic and Non-Communicable Disease Control and Prevention, Chinese Center for Disease Control and Prevention, Beijing, 100050 China

**Keywords:** Tuberculosis, Spatial-temporal, Associated factors, Meteorological factors, Inner Mongolia

## Abstract

**Background:**

Tuberculosis (TB) is an important public health issue worldwide. However, evidence concerning the impact of environmental factors on TB is sparse. We performed a retrospective analysis to determine the spatiotemporal trends and geographic variations of, and the factors associated with, the TB prevalence in Inner Mongolia.

**Methods:**

We performed a retrospective analysis of the epidemiology of TB. A Bayesian spatiotemporal model was used to investigate the spatiotemporal distribution and trends of the TB prevalence. A spatial panel data model was used to identify factors associated with the TB prevalence in the 101 counties of Inner Mongolia, using county-level aggregated data collected by the Inner Mongolia Center for Disease Control and Prevention.

**Results:**

From January 2010 to December 2014, 79,466 (6.36‱) incident TB cases were recorded. The TB prevalence ranged from 4.97‱ (12,515/25,167,547) in 2014 to 7.49‱ (18,406/ 24,578,678) in 2010; the majority of TB cases were in males, and in those aged 46–60 years; by occupation, farmers and herdsmen were the most frequently affected. The Bayesian spatiotemporal model showed that the overall TB prevalence decreased linearly from 2010 to 2014 and occupation-stratified analyses yielded similar results, corroborating the reliability of the findings. The decrease of TB prevalence in the central-western and eastern regions was more rapid than that in the overall TB prevalence. A spatial correlation analysis showed spatial clustering of the TB prevalence from 2011 to 2014 (Moran’s index > 0, *P* < 0.05); in the spatial panel data model, rural residence, birth rate, number of beds, population density, precipitation, air pressure, and sunshine duration were associated with the TB prevalence.

**Conclusions:**

The overall TB prevalence in Inner Mongolia decreased from 2010 to 2014; however, the incidence of TB was high throughout this period. The TB prevalence was influenced by a spatiotemporal interaction effect and was associated with epidemiological, healthcare, and environmental factors.

**Electronic supplementary material:**

The online version of this article (10.1186/s12879-019-3910-x) contains supplementary material, which is available to authorized users.

## Background

Tuberculosis (TB) is a chronic respiratory infectious disease caused by *Mycobacterium tuberculosis* (MTB) [1]. According to the World Health Organization, approximately 1.7 billion (23%) people have latent TB infections and an estimated 10.0 million (range, 9.0–11.1 million) new cases of TB are reported annually. Two-thirds of these cases occur in eight countries: India (27%), China (9%), Indonesia (8%), the Philippines (6%), and Pakistan (5%), Nigeria (4%), Bangladesh (4%) and South Africa (3%). In addition, TB caused an estimated 1.3 million (range, 1.2–1.4 million) deaths in 2017. Notably, China has the fourth highest number of incident TB cases, and second number of multidrug resistant TB cases in 30 high‑burden countries worldwide [2]. Thus, TB is a critical public health challenge worldwide, particularly in low- and middle-income countries.

The Fifth Chinese Epidemiological Survey in 2010 [3] showed that the prevalences of active TB, smear-positive TB, and bacteriologically positive TB in western China were 695/100,000, 105/100,000, and 198/100,000, respectively; these numbers are higher than those for the central and eastern regions, but lower than that for China as a whole. Inner Mongolia is located in northern China, and has up to 200,000 TB cases; about 3,500 people in Inner Mongolia die from TB annually. In the 2011 National Surveillance of TB Report [4], the prevalences of active TB, smear-positive TB, and bacteriologically positive TB in Inner Mongolia were 757/100,000, 78/100,000, and 183/100,000, respectively; these numbers are higher than the national average. The factors associated with TB are of interest to healthcare personnel responsible for the care of high-risk patients, and include sociodemographic [5, 6] and genetic [7, 8] factors. However, the effects of environmental factors [9–11], particularly those related to meteorology, on the TB prevalence are typically overlooked.

Geographic information systems have been used in studies of infectious diseases, *e*.*g*., to detect hot spots and epidemics [12, 13]. However, assessment of the effects of various factors on the TB prevalence is hampered by the dearth of data on incident cases in Inner Mongolia. Therefore, we investigated the spatiotemporal trends and geographic variations of the TB prevalence in Inner Mongolia from 2010 to 2014 and identified related meteorological factors.

## Methods

### Data Sources

Data on all TB cases reported from January 2010 to December 2014 in the 101 districts and counties of Inner Mongolia were obtained from the communicable disease surveillance system of the Inner Mongolia Center for Disease Control and Prevention (CDC). County-year demographic data were provided by the Inner Mongolia Bureau of Statistics and meteorological data (humidity, air pressure, precipitation, sunshine duration, and wind speed) were obtained from the Inner Mongolia Meteorological Office Sharing Service System. Digital maps were generated from geographical boundary data at the prefecture level (Chinese CDC). The annual TB prevalence was calculated by summing the county-level prevalences, with weighting of each county-level prevalence by the population of that county. In other words, the prevalence was calculated as:

$$ \sum \frac{\mathrm{n}}{\mathrm{i}=1}\left(w\mathrm{i}\times TB\mathrm{prevalencei}\right)/\sum \frac{\mathrm{n}}{\mathrm{i}=1}w\mathrm{i}\times 100000/100000, $$

where *n* is the number of counties, *w*_*i*_ is the population of the *i*^*th*^ county, and *TBprevalence*_*i*_ is the TB prevalence in the *i*^*th*^ county.

#### Statistical Analyses

### Spatial Autocorrelation Analysis

A global spatial autocorrelation analysis using Moran’s index (Moran's *I*) was performed to assess the spatial clustering of TB prevalences in Inner Mongolia. The significance of Moran’s *I* is identified based on the Monte Carlo method, which simulates the distribution according to random data derived from Pearson’s correlation coefficients [14]. Local spatial autocorrelation was used to detect high- and low- risk areas for the TB prevalence. A Moran’s *I* value > 0 indicates that the TB prevalence of district *i* and its neighboring districts differs significantly from that of other districts. District *i* is the center of the area with the higher/ lower TB prevalence, and is defined as a high-high/ low-low area for TB prevalence, respectively. In contrast, the TB prevalence between districts shows a discrete distribution at Moran’s *I* < 0 and a random distribution at Moran’s *I* = 0.

#### Bayesian Spatiotemporal Modeling

Bayesian spatiotemporal modeling was conducted to investigate the spatiotemporal trends of the TB prevalence in Inner Mongolia. Bayesian spatiotemporal modeling can involve a combination of the common spatial pattern and the common time trend (to represent the stable component of the TB risk); or spatiotemporal interactions, with the pattern partitioned into local (borough-level) time trends and a random error that affects the dependent variable not included in the model.

For modeling of rare TB cases, Poisson regression with the log-link function is typically used. We let y_it_ and n_it_ be the number of incident TB cases and the population, respectively, in area *I* = (1, …, 101) at timepoint *t* = (1, …, 5). Incident TB cases can be modeled as:

$$ \mathrm{yit}\sim \mathrm{Poisson}\left(\mathrm{ni}\ \mu \mathrm{it}\right). $$

The underlying TB risk U_it_ is modeled as:

$$ \log \left(\mu \mathrm{it}\right)=\alpha + Si+b\mathrm{o}{t}^{\ast }+\upsilon t+b1{it}^{\ast }+\varepsilon it, $$

where t* = *t* − 2.5 (cantering at the mid-over period) and *α* is the overall TB risk for the 5‑year study period in Inner Mongolia. (**S***i* + *b*ot^∗^ + *υ*t) is the common spatiotemporal pattern that represents the stable component of the TB risk across the 5 years. The spatial term **S***i*, which is common to all 5 years, describes the spatial distribution of TB risk across Inner Mongolia. (*b*ot^∗^ + *υt*) represents the overall time trend common to all counties. *b*ot^∗^ represents a linear trend, and *υ*t allows for nonlinearity in the overall trend pattern. *b*1*i*t^∗^ + *εit* represents the spatiotemporal interactions, and *b*1*i*t^∗^ allows each borough to have its own trend. *εit* is the variability in the data not explained by the other components of the model.

#### Spatial Panel Data Model

The TB prevalence in each county (as the dependent variable) and demographic and meteorological variables were included in the spatial panel data model to identify spatiotemporal factors associated with the TB prevalence. The classic Hausman test is typically used to identify individual effects in a panel data model. Independent variables that do not change over time do not affect the dependent variable, indicating that the model has a fixed effect; in contrast, the model has a random effect. The spatial panel data model can be decomposed into a spatial lag model (SLM) and spatial error model (SEM) according to the spatial correlation, and the more suitable of the two models can be selected using the Lagrange multiplier (LM) test. The classic spatial panel data model used in this study was:

$$ {\displaystyle \begin{array}{l}y\mathrm{it}=\rho {\mathrm{Wn}}^{\ast}\mathrm{n}\mathrm{yit}+\beta X+\mu \\ {}\mu =\lambda {\mathrm{Wn}}^{\ast}\mathrm{n}\mu +\varepsilon .\end{array}} $$

The SEM was adopted when *ρ* = 0, indicating that the spatial dependency of the dependent variable led to spatial autocorrelation in the dependent lag. The SLM was selected when *λ* = 0, indicating that spatial dependency leading to spatial autocorrelation exists in the error term of the dependent variable.

In the above equation, *X* and *y* are observations and *i* and *t* are the county and timepoint, respectively. *ρ*Wn^∗^n yit is the spatial-dependent lag. *ρ* is the spatial autocorrelation coefficient, a measure of the effect of Wn^∗^nyit on yit. Wn^∗^n is the spatial weight matrix, *β* is the spatial autoregressive coefficient, and *ε* is the spatial error term. *μ* is the disturbance in spatial dependency and *λ* is the spatial autocorrelation coefficient [15].

Statistical analysis was performed using SPSS 19.0, and two-sided *P* values < 0.05 were regarded as indicative of statistical significance. Geographic analysis was performed using OpenBUGS 3.2.3 and ArcGIS 10.2 (Desktop Advanced, Student Edition). The multivariate analysis was conducted using R 3.3.2. The Econometric Models for Spatial Panel Data (splm) package in R 3.3.2 was used for the spatial panel analysis (https://www.r-project.org/).

## Results

### Descriptive Analysis

#### Trends of the TB Prevalence According to Sex and Age

From 2010 to 2014, the TB prevalence among males and females decreased markedly in Inner Mongolia. The 3.01% decrease in the TB prevalence among males during this period included a 1.43% decrease from 2012–2013. The 1.83% decrease in the TB prevalence among females during this period included a 0.69% decrease from 2013–2014. The TB prevalence was higher among males than among females (Additional file 1: Figure S1). In males and females, TB cases were concentrated in those aged 46–60 years (27.3%, 28.2%, 29.7%, 28.3%, and 28.7%, respectively, from 2010 to 2014; Additional file 2: Table S1).

#### TB Prevalence According to Occupation

The occupations with the top five frequencies of incident TB were farmers and herdsmen (65.9%), unemployed (11.2%), other (6.8%), students (4.7%), and retirees (3.3%); together, these accounted for 92% of the total. Moreover, incident TB cases were concentrated in farmers and herdsmen in each of the 5 years of the study (Additional file 3: Figure S2).

### Temporal Trends in the TB Prevalence in Inner Mongolia

**Fig. 1:**
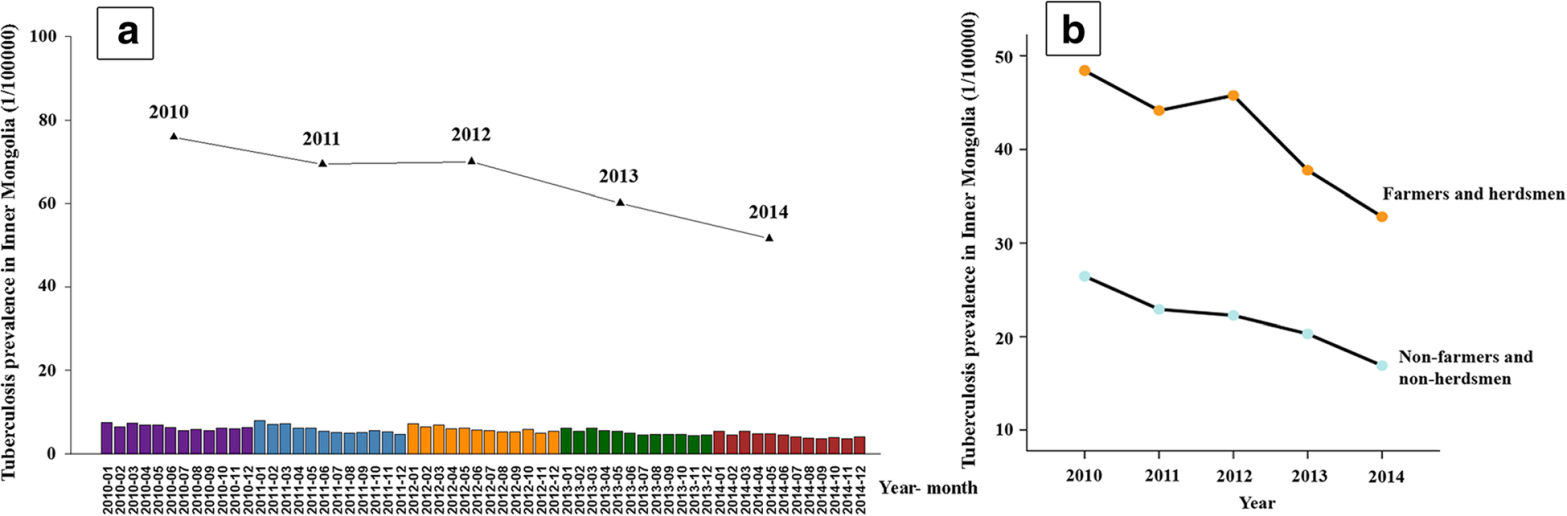
Temporal trends in the TB prevalence in Inner Mongolia, 2010–2014. Trend of the overall TB prevalence (**a**) and of the TB prevalence according to occupation (**b**)

In Inner Mongolia from 2010 to 2014, the incident and overall TB prevalences were 79,466 and 6.36, respectively; the annual TB prevalences were 7.49‱ (18,406/24,578,678), 6.71‱ (16,865/25,145,774), 6.80‱ (17,076/25,103,548), 5.81‱ (14,603/25,145,585), and 4.97‱ (12,515/25,167,547), respectively (Fig. 1a). The overall TB prevalence decreased during the study period; the decrease was linear from 2012 to 2014. The TB prevalence was highest from January to May. The TB prevalence among farmers and herdsmen, as well as that among non-farmers and non-herdsmen, decreased markedly during the study period (*P* for trend < 0.001; Fig. 1b). The average annual TB prevalence decreased by 3.2% for farmers and herdsmen and 2.0% for non-farmers and non-herdsmen from 2010 to 2014; however, the TB prevalence among farmers and herdsmen remained high throughout the study period.

### Geographic Variation in the TB Prevalence

#### Distribution of TB Prevalences

**Fig. 2:**
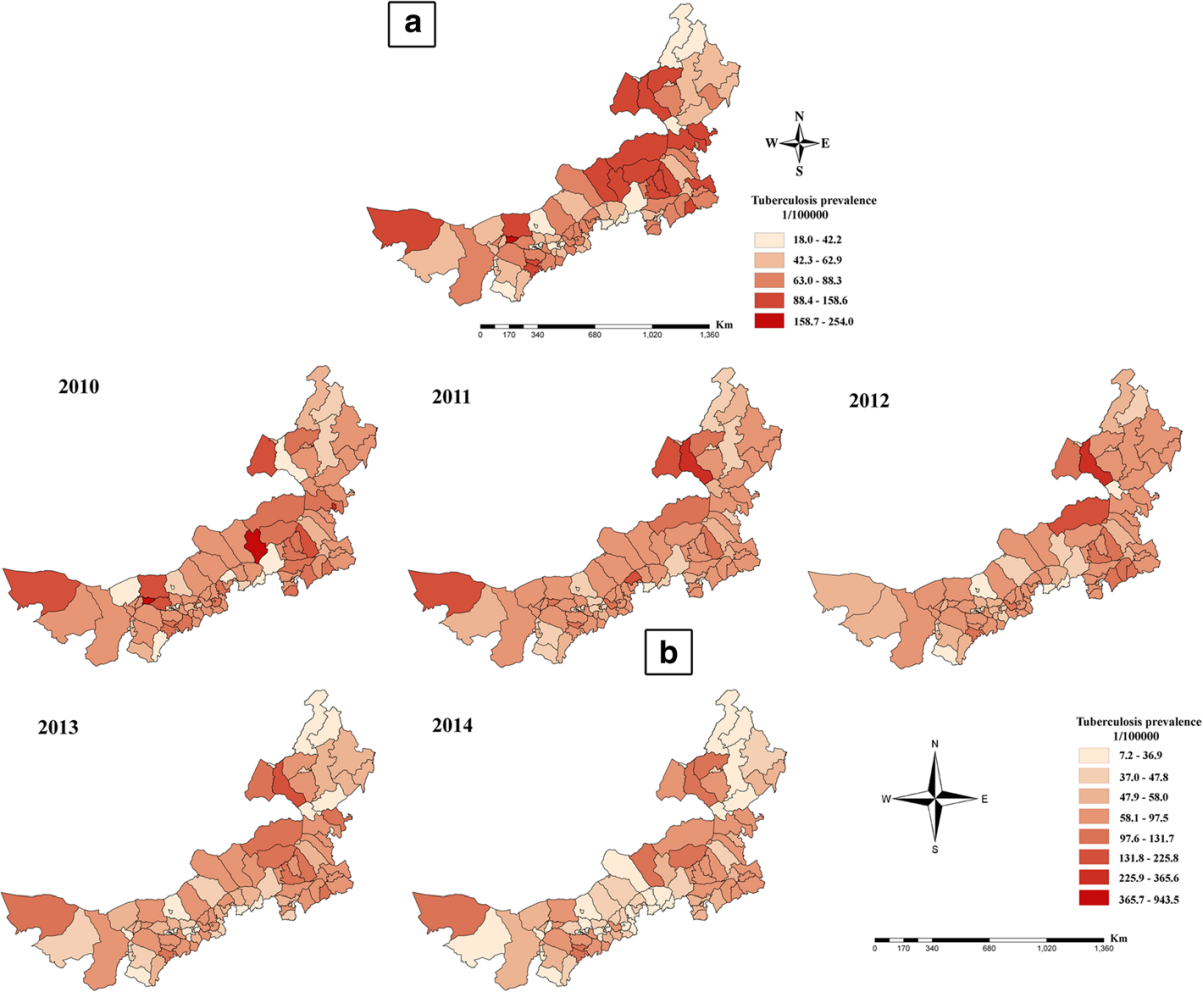
Spatial distribution of the TB prevalence in Inner Mongolia, 2010–2014. Spatial distribution of overall TB cases by county over 5 years (**a**) and of the TB prevalence by county (**b**)

The overall TB prevalence was high mainly in the central and eastern regions of Inner Mongolia from 2010 to 2014; the TB prevalence in the western region was lower (Fig. 2a). TB spread from the central to the eastern and western regions of Inner Mongolia (Fig. 2b). At the county level, the highest TB prevalences from 2010 to 2014 were 94.35‱ (27/17,249) for Wuyuan of Bayan Nur, 25.64‱ (107/41,728) for New Barag Left Banner of Hulun Buir, 23.47‱ (99/42,178) for New Barag Left Banner of Hulun Buir, 14.32‱ (61/42,592) for New Barag Left Banner of Hulun Buir, and 13.08‱ (59/45,116) for Abaga Banner of XilinGol League. The lowest TB prevalences were 1.38‱ (47/341,641) for Wulatehou Banner of Bayan Nur, 1.92‱ (94/489,900) for Qingshan of Baotou, 1.47‱ (31/211,401) for Taipusi Banner of the XilinGol League, 1.13‱ (57/504,200) for Qingshan of Baotou, and 0.72‱ (8/110,348) for Duolun of the XilinGol League (Fig. 2b).

**Fig. 3:**
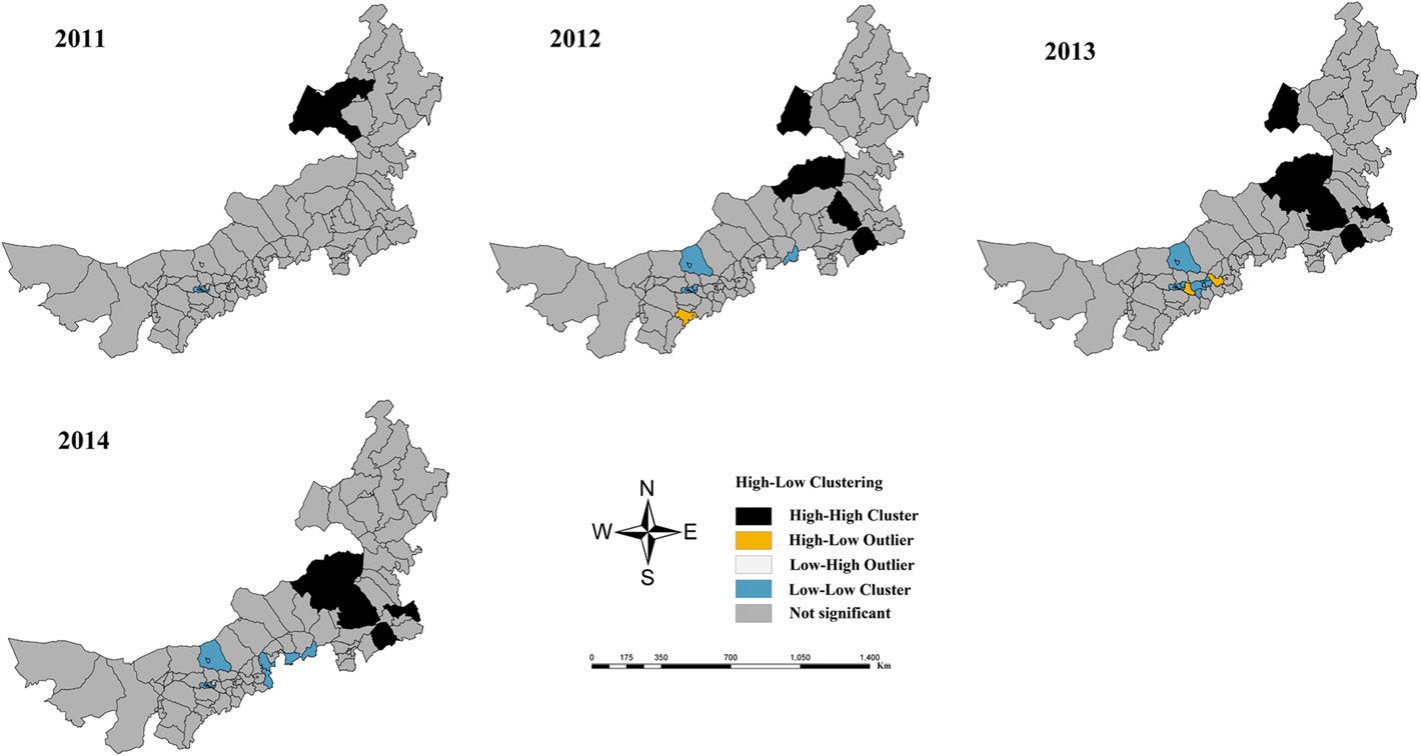
Local spatial autocorrelation of the TB prevalence in Inner Mongolia, 2010–2014

The TB prevalences showed marked geographic clustering from 2011 to 2014 (*P* < 0.001, Monte Carlo test; Table 1). Moran’s *I* values for global spatial autocorrelation were 0.181, 0.213, 0.249, and 0.251 for 2011 to 2014, respectively. The local spatial autocorrelation of the TB prevalence showed that high-high areas were concentrated in Hulun Buir in 2011; Hulun Buir, the XilinGol League, Chifeng, and Tongliao in 2012 and 2013; and the XilinGol League, Chifeng, and eastern areas of Tongliao in 2014, suggesting that the high-high areas spread from the northeastern to the southeastern region of Inner Mongolia (Fig. 3).

**Table 1:** The Global spatial autocorrelation and trend in TB prevalence in Inner Mongolia, 2010-2014

Year	Moran’s *I*	Var(*I*)	*Z*	*P*
2010	0.015	0.001	0.651	0.515
2011	0.181	0.003	4.081	<0.001*
2012	0.213	0.003	3.531	<0.001*
2013	0.249	0.003	4.540	<0.001*
2014	0.251	0.003	4.585	<0.001*

*P<0.001

#### Spatiotemporal Trends in the TB Prevalence

**Fig. 4:**
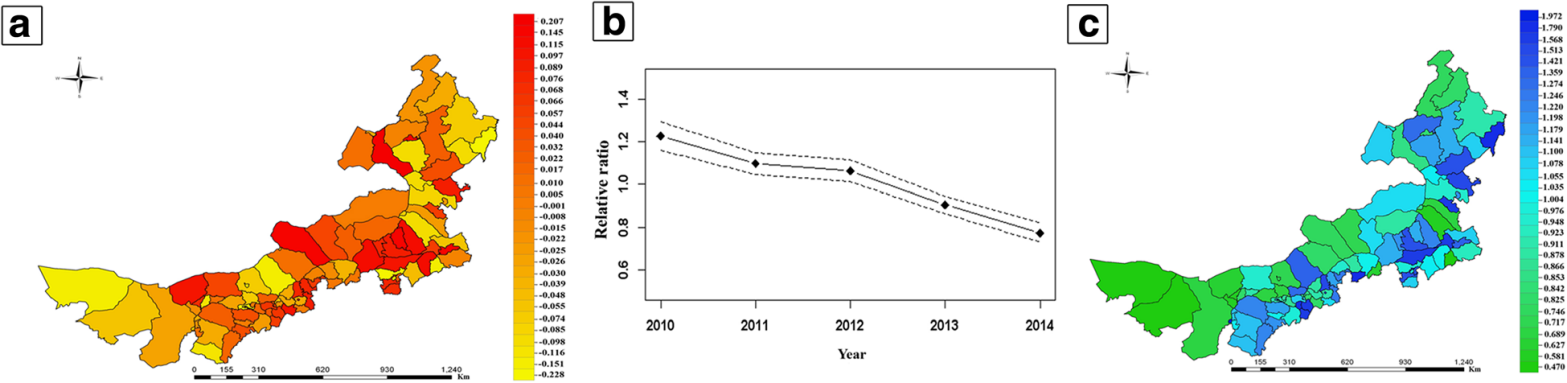
Bayesian spatiotemporal modeling of the TB prevalence in Inner Mongolia. Estimated local variations (**a**), common temporal trend (**b**), and estimated common spatial pattern (**c**)

Figure 4 shows the estimated spatiotemporal variations in the TB prevalence. Compared with that in the overall TB prevalence from 2010 to 2014, the decrease in the TB prevalence was considerably more rapid in Alxa Right Banner of Alxa, Wuyuan of Bayan Nur, Zhalantun of Hulun Buir, Ulanhot of Hinggan League, Taipusi Banner of XilinGol League, and Qingshan of Baotou; the prevalence of decrease was slower in other areas (Fig. 4a). The TB prevalence showed a significant decreasing trend over time (Fig. 4b). The following areas had high TB prevalences: Qingshuihe of Hohhot and adjacent central and western areas, Wengniute of Chifeng and adjacent northwestern areas, and Daur Autonomous Banner of Morin Dawa in Hulun Buir and adjacent northwestern areas (Fig. 4c).

#### Factors Associated with the TB Prevalence

In Hausman and LM tests, no random effect was detected in the spatial panel data model and the autocorrelation comprised mainly the lag term (*χ*^2^ = 33.41, *P* < 0.005; LM = 0.0052, *P* = 0.10); thus, the spatial panel lag model with fixed effects was used. The spatial panel data model comprised six sociodemographic variables and five meteorological variables, with the monthly average TB prevalence serving as the dependent variable. The TB prevalence was associated positively with rural residence (*B* = 0.044), birth prevalence (*B* = 0.060), air pressure (*B* = 0.024), and sunshine duration (*B* = 0.022), and negatively with the number of beds (*B* = −0.031), population density (*B* = −0.021), and precipitation (*B* = −0.022, *P* < 0.001; Table 2).

**Table 2:** Association of associated factors with TB prevalence in Inner Mongolia, 2010-2014

Variable	*B*	Standard Error	*t*	*P*
Proportion of male	-0.011	0.007	-1.673	0.094
Rural residence	0.044	0.010	4.600	<0.001*
Birth rate	0.060	0.008	7.204	<0.001*
Average gross domestic product	0.012	0.009	1.326	0.185
Number of beds	-0.031	0.010	-3.209	<0.001*
Population density	-0.021	0.011	-1.989	<0.001*
Precipitation	-0.022	0.006	-3.473	<0.001*
Air pressure	0.024	0.007	3.457	<0.001*
Sunshine duration	0.022	0.006	3.418	<0.001*
Humidity	0.000	0.006	0.078	0.938
Wind speed	0.001	0.006	0.216	0.829
Intercept	0.383	0.015	25.971	<0.001*

*P<0.001

## Discussion

Inner Mongolia is a critical area in China for TB prevention and control due to its poor conditions, backward economy, multi-ethnic settlements, and ongoing TB epidemic [16]. MTB is generally transmitted via the airborne route, with association between cases and epidemic interaction between neighboring areas [17, 18], meaning that areas close to high-epidemic areas are at elevated risk of TB epidemics.

In this study, the TB prevalence was higher among males than among females, possibly due to the former being at greater risk of exposure to MTB as a result of differences in socioeconomic and cultural factors. In addition, males frequently smoke tobacco and drink alcohol; the latter may lead to liver damage [19], increasing the risk of MTB infection. The TB prevalence was highest in those aged 46–60 years, likely due to age-related degeneration of physical function and T‑cell–mediated immune dysfunction [20], consistent with prior reports [21, 22]. Additionally, farming was the occupation at greatest risk of TB. Rural areas typically have poor economic conditions and scarce public health services, particularly in the absence of community resources such as roads, water, and electricity [23]. MTB is inactivated under dry, sunny conditions; in contrast, humidity and poor ventilation increase the risk of TB [24].

The birth prevalence was associated positively with the TB prevalence. Children, and particularly infants, have weak cellular immunity and immature lungs, which increases their risk of MTB infection [25]. In addition, MTB can be transmitted to infants by family members and neighbors [26]. The TB prevalence among children aged 0–14 years was 0.005%, lower than both that reported by the Fourth TB Epidemiological Survey in China and the prevalences in other high-TB‑burden areas, *e*.*g*., Mexico (7.8–32.0%) [27], Turkey (3.4%), and other middle-income countries [28]. In Inner Mongolia, the TB prevalence is lower in children than in adults, but TB in children tends to be atypical, progress rapidly, and disseminate readily [29].

Population density and the number of beds were associated negatively with the TB prevalence. The TB prevalence was higher in several undeveloped areas, which have poor living environment, a dearth of human resources, greater dispersal of residents, road transportation problems, and insufficient medical resources [30]. A low-rise, high-density residential environment is associated negatively with the TB prevalence [31]. Residents of high-rise buildings benefit from better ventilation and more direct sunlight, which reduce the risk of MTB infection. In contrast, the low air quality and little sunshine experienced by residents of impoverished areas increase the risk of TB, which may explain the positive association between rural residence and the TB prevalence. Most rural areas are underdeveloped with low population density and limited number of beds, and the associated low income, educational level, and awareness of health-related issues facilitate the spread of TB [31–33].

The impact of meteorological factors on human health, and particularly infectious disease, is of interest worldwide [34, 35]. Sunshine duration and air pressure were associated positively with the TB prevalence, consistent with previous reports [36, 37]. Humidity tends to decrease and temperature to increase with increasing sunshine duration. Furthermore, air conditioning in rooms with limited ventilation facilitates the spread of MTB [38]. The continental climate of Inner Mongolia is dry and arid, and the air pressure during winter and spring is higher than that in summer. In this study, the TB prevalence was higher in winter and spring, suggesting a positive correlation with air pressure. This may be because high air pressure enhances atmospheric flow, promoting the transmission of MTB [11]. Precipitation was associated negatively with the TB prevalence, possibly because rain tends to increase the air quality and reduce the MTB concentration [39]. Therefore, prevention and management of TB should be strengthened in areas that receive little precipitation.

Our study has several strengths. To our knowledge, it is the largest study of the trend of, and factors associated with, the TB prevalence in Inner Mongolia from 2010 to 2014. Moreover, we estimated the TB prevalence according to demographic characteristics, *e*.*g*., age and sex. However, this study also has several limitations. First, it was a retrospective analysis; a prospective study would have enabled prediction of the trend of the TB prevalence. Second, aggregation of individual cases to the district level may have resulted in individual differences being missed; however, we assessed the effects of multiple factors on the TB prevalence in each district. Finally, the survey covered only certain regions in China, which hampers extrapolation of our findings.

## Conclusions

The TB prevalence in Inner Mongolia decreased significantly from 2010 to 2014, and was highest in the central-eastern and western regions. Our findings suggest that several often‑overlooked meteorological factors are associated with a high TB prevalence. Moreover, our data support the need for implementation of prospective surveillance of TB transmission, particularly in low- and middle-income regions and those in which public health services are scarce.

## Additional files

Additional file 1: **Figure S1.** TB prevalence in Inner Mongolia according to sex, 2010–2014. (TIF 7326 kb)

Additional file 2: **Table S1.** Number of TB cases in Inner Mongolia according to sex and age, 2010–2014. (DOC 48 kb)

Additional file 3: **Figure S2.** TB prevalence in Inner Mongolia according to occupation, 2010–2014. (TIF 7296 kb)

## Abbreviations

GDP: Gross Domestic Product
GIS: Geographic Information System
LM test: Lagrange multiplier test
Moran's *I*: Moran's index
MTB: Mycobacterium tuberculosis
SEM: Spatial Error model
SLM: Spatial Lag model
TB: Tuberculosis
