# Supplementary figures and images for: Spatiotemporal epidemiology of, and factors associated with, the tuberculosis prevalence in northern China, 2010–2014
Source: BMC Infect Dis. 2019 Apr 30;19:365. doi: 10.1186/s12879-019-3910-x (PMC6492399; doi:10.1186/s12879-019-3910-x)

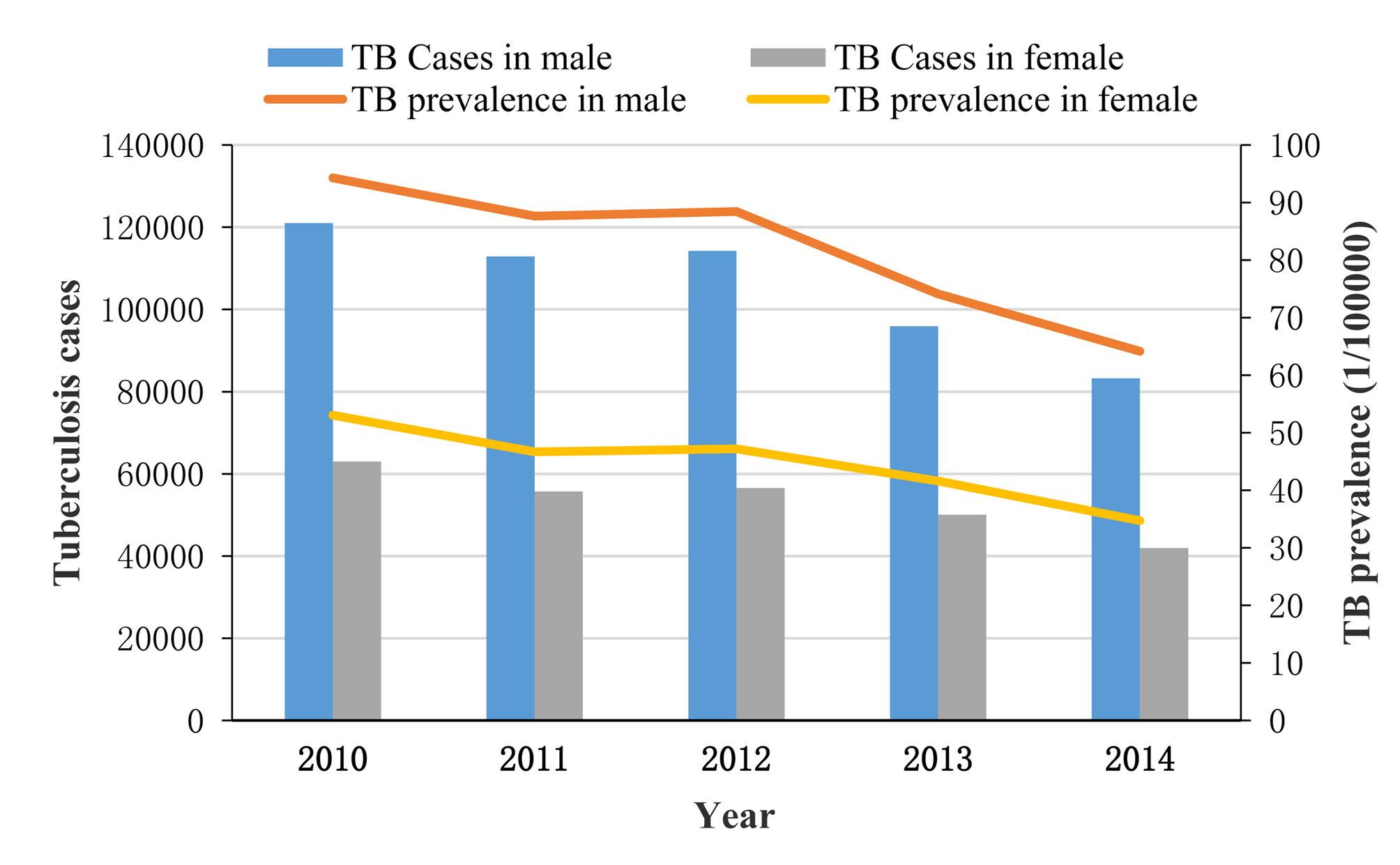

Supplement: Supplementary file 1 — Figure S1. TB prevalence in Inner Mongolia according to sex, 2010–2014. (TIF 7326 kb) [file 12879_2019_3910_MOESM1_ESM.tif]

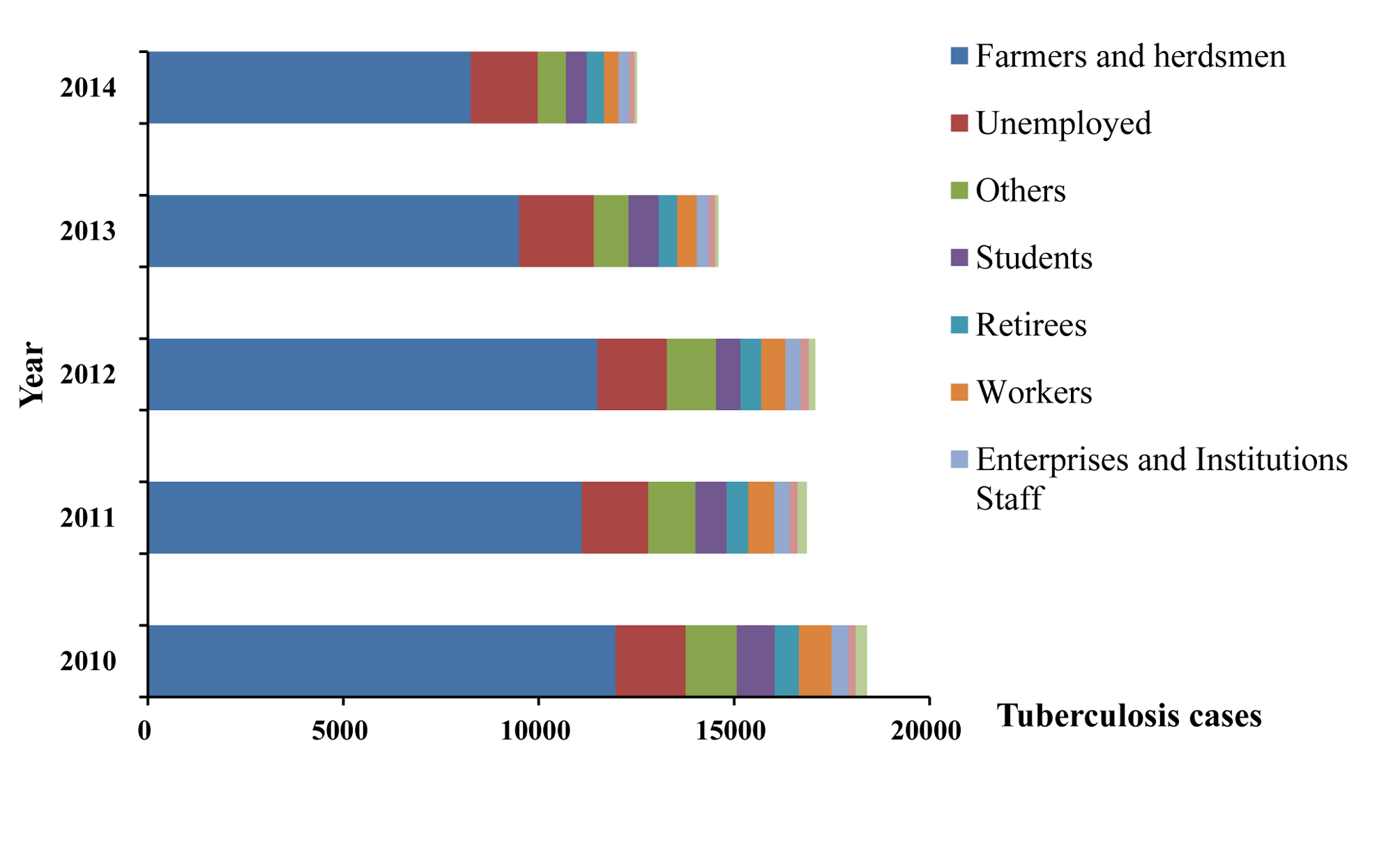

Supplement: Supplementary file 3 — Figure S2. TB prevalence in Inner Mongolia according to occupation, 2010–2014. (TIF 7296 kb) [file 12879_2019_3910_MOESM3_ESM.tif]
