# Supplementary material for: Spatiotemporal epidemiology of, and factors associated with, the tuberculosis prevalence in northern China, 2010–2014
Source: BMC Infect Dis. 2019 Apr 30;19:365. doi: 10.1186/s12879-019-3910-x (PMC6492399; doi:10.1186/s12879-019-3910-x)
Supplement: Supplementary file 2 — Table S1. Number of TB cases in Inner Mongolia according to sex and age, 2010–2014. (DOC 48 kb) [file 12879_2019_3910_MOESM2_ESM.doc]

Table S1. The TB cases across sex-age in Inner Mongolia, 2010-2014.

| Year | 0- | | 16- | | | 31- | | | 46- | | | 61- | | | 76- | | |
| --- | --- | --- | --- | --- | --- | --- | --- | --- | --- | --- | --- | --- | --- | --- | --- | --- | --- |
| 2010 |  |  | |  |  | |  |  | |  |  | |  |  | |  |  |
| Male | 110(0.1) |  | | 2114(17.6) |  | | 2837(23.5) |  | | 3510(29.0) |  | | 2690(22.2) |  | | 833(6.9) |  |
| Female | 72(1.1) |  | | 1532(24.3) |  | | 1332(21.2) |  | | 1518(24.1) |  | | 1397(22.2) |  | | 446(7.1) |  |
| 2011 |  |  | |  |  | |  |  | |  |  | |  |  | |  |  |
| Male | 45(0.4) |  | | 2005(17.8) |  | | 2627(23.3) |  | | 3361(29.8) |  | | 2418(21.4) |  | | 822(7.3) |  |
| Female | 46(0.8) |  | | 1332(23.9) |  | | 1130(20.3) |  | | 1392(25.0) |  | | 1241(22.3) |  | | 434(7.8) |  |
| 2012 |  |  | |  |  | |  |  | |  |  | |  |  | |  |  |
| Male | 44(0.4) |  | | 1844(16.1) |  | | 2606(22.8) |  | | 3600(31.5) |  | | 2484(21.8) |  | | 840(7.4) |  |
| Female | 43(0.8) |  | | 1233(21.8) |  | | 1199(21.2) |  | | 1464(25.9) |  | | 1286(22.8) |  | | 427(7.6) |  |
| 2013 |  |  | |  |  | |  |  | |  |  | |  |  | |  |  |
| Male | 39(0.4) |  | | 1797(18.7) |  | | 2105(21.9) |  | | 2875(30.0) |  | | 2039(21.2) |  | | 741(7.7) |  |
| Female | 43(0.9) |  | | 1134(22.6) |  | | 1046(20.9) |  | | 1257(25.1) |  | | 1091(21.8) |  | | 436(8.7) |  |
| 2014 |  |  | |  |  | |  |  | |  |  | |  |  | |  |  |
| Male | 24(0.3) |  | | 1416(17.0) |  | | 1805(21.7) |  | | 2554(30.7) |  | | 1785(21.5) |  | | 736(8.8) |  |
| Female | 29(0.7) |  | | 945(22.5) |  | | 799(19.1) |  | | 1037(24.7) |  | | 987(23.5) |  | | 397(9.5) |  |
